# Serendipitous Discovery of a Competitive Inhibitor of FraB, a *Salmonella* Deglycase and Drug Target

**DOI:** 10.3390/pathogens11101102

**Published:** 2022-09-26

**Authors:** Pankajavalli Thirugnanasambantham, Sravya Kovvali, Austin Cool, Yuan Gao, Anice Sabag-Daigle, Erin F. Boulanger, Mark Mitton-Fry, Angela Di Capua, Edward J. Behrman, Vicki H. Wysocki, Steffen Lindert, Brian M. M. Ahmer, Venkat Gopalan

**Affiliations:** 1 Department of Chemistry and Biochemistry, The Ohio State University, Columbus, OH 43210, USA; 2 Department of Microbiology, The Ohio State University, Columbus, OH 43210, USA; 3 Resource for Native Mass Spectrometry-Guided Structural Biology, The Ohio State University, Columbus, OH 43210, USA; 4 Department of Microbial Infection and Immunity, The Ohio State University, Columbus, OH 43210, USA; 5 Department of Medicinal Chemistry and Pharmacognosy, The Ohio State University, Columbus, OH 43210, USA

**Keywords:** *Salmonella* deglycase, drug discovery, competitive inhibitor, serendipity

## Abstract

Although salmonellosis, an infectious disease, is a significant global healthcare burden, there are no *Salmonella*-specific vaccines or therapeutics for humans. Motivated by our finding that FraB, a *Salmonella* deglycase responsible for fructose-asparagine catabolism, is a viable drug target, we initiated experimental and computational efforts to identify inhibitors of FraB. To this end, our recent high-throughput screening initiative yielded almost exclusively uncompetitive inhibitors of FraB. In parallel with this advance, we report here how a separate structural and computational biology investigation of FrlB, a FraB paralog, led to the serendipitous discovery that 2-deoxy-6-phosphogluconate is a competitive inhibitor of FraB (K_I_ ~ 3 μM). However, this compound was ineffective in inhibiting the growth of *Salmonella* in a liquid culture. In addition to poor uptake, cellular metabolic transformations by a *Salmonella* dehydrogenase and different phosphatases likely undermined the efficacy of 2-deoxy-6-phosphogluconate in live-cell assays. These insights inform our ongoing efforts to synthesize non-hydrolyzable/-metabolizable analogs of 2-deoxy-6-phosphogluconate. We showcase our findings largely to (re)emphasize the role of serendipity and the importance of multi-pronged approaches in drug discovery.

## 1. Introduction

Non-typhoidal serovars of *Salmonella* result in intestinal infection and gastroenteritis in humans. *Salmonella enterica* serovar Typhimurium is a leading cause of food borne illness in the United States and globally. To date, there are no vaccines or narrow-spectrum antibiotics available to protect humans from the acute inflammatory diarrhea and fever that result upon infection by this bacterial pathogen. Thus, identification of new drug targets in *Salmonella* and characterization of their inhibitors is a high research priority. This overarching objective is the motivation for this study.

Many bacteria and fungi can grow on fructosamines, which are generated following an Amadori rearrangement of the Schiff base that is formed when an aldose (e.g., glucose) reacts with a primary amine (e.g., the alpha-amino group in an amino acid). The degradation of fructosamines typically entails either oxidases or the combined use of a kinase and deglycase [1,2,3] (Supplementary Figure S1). We established that the latter route is used by *Salmonella* when it exploits fructose-asparagine (F-Asn), an Amadori compound, as a carbon and nitrogen source during growth in the inflamed intestine [4]. We have validated a three-step pathway for the utilization of F-Asn using enzymes encoded by the *fra* locus in *Salmonella* [5,6,7] (Supplementary Figure S1). FraE, a periplasmic asparaginase, converts F-Asn to F-Asp, whose uptake is followed by a kinase (FraD)-mediated phosphorylation to 6-phosphofructose-aspartate (6-P-F-Asp). This sugar-phosphate in turn is converted by FraB, a deglycase, to glucose-6-phosphate and L-aspartate, two central metabolic intermediates. Surprisingly, disrupting *fraB* results in intoxication due to an accumulation of 6-P-F-Asp and causes dramatic attenuation in mouse models [5]. These findings inspired us to evaluate the prospects of FraB as a drug target.

We recently conducted a high-throughput screen (HTS) to identify FraB inhibitors using cell-based and biochemical assays [8]. This initiative led to identification of a few hits, which we are now pursuing further using medicinal chemistry approaches. Unexpectedly, the HTS undertaking has thus far yielded near exclusively uncompetitive inhibitors [8]. Our parallel efforts, however, to gain a broad understanding of bacterial metabolism of Amadori compounds led to the fortuitous discovery of a competitive inhibitor of FraB. This outcome is the focus of this report.

Several bacteria including *Salmonella*, *Bacillus subtilis,* and *Escherichia coli* [2,3,9] can metabolize F-Lys (an ε-conjugated Amadori product unlike F-Asn, which is α-conjugated). The final catabolic step for F-Lys utilization involves FrlB deglycase, which converts 6-phosphofructose-lysine (6-P-F-Lys) to glucose-6-phosphate and L-lysine (Supplementary Figure S1). Since FraB and FrlB are specific for their respective substrates [unpublished data], we have been interested in uncovering the molecular basis for this discrimination especially since the two enzymes employ a conserved general acid and general base to facilitate bond breaking [6,10]. Therefore, we aimed to obtain crystal structures of these two deglycases with and without their respective substrates. Although we have high-resolution structures of FraB and FrlB [unpublished data], we are yet to co-crystallize either enzyme with its respective substrate. As an alternative, we sought to identify and use non-cleavable substrate analogs that may yield stable, crystallizable ES-type complexes. To this end, we searched the National Cancer Institute (NCI) Open Database Compounds [11] for molecules similar to either 6-P-F-Asp or 6-P-F-Lys. One of the compounds identified by querying with 6-P-F-Lys predictably inhibited FrlB, but it unexpectedly showed a stronger inhibition of FraB. This chance finding provides a basis for design and development of one class of FraB inhibitors.

## 2. Results

### 2.1. Tanimoto Search of the NCI Database for Small Molecules Based on Structural Similarity

**Figure 1:**
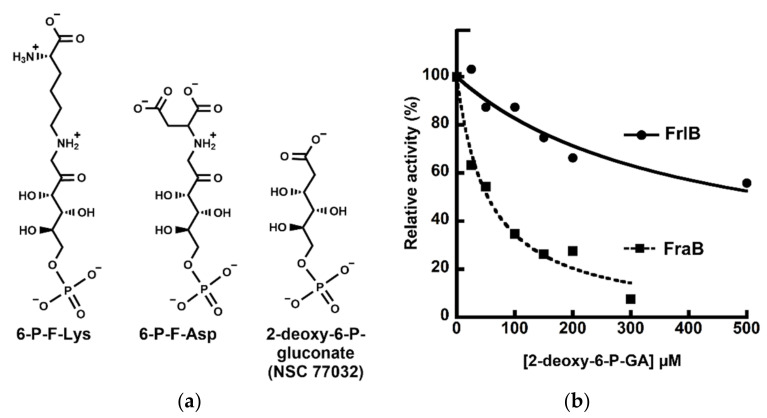
(**a**) Structures of 6-P-F-Lys and 6-P-F-Asp, the substrates of FrlB and FraB, respectively, and of 2-deoxy-6-P-gluconate (2-deoxy-6-P-GA), the inhibitor that is the mainstay of this study. (**b**) Representative IC_50_ curves from one of the two trials (data for the second trial are shown in Supplementary Figure S3). Plots indicate relative activity of FraB (squares) or FrlB (circles) in the presence of increasing concentrations of 2-deoxy-6-P-GA. The goodness of fit values are 0.99 and 0.98 for the FraB and FrlB IC_50_ curves, respectively.

We applied the Tanimoto index available in the RDKit to evaluate the structural similarity of 6-P-F-Lys (the substrate of FrlB) to any of the 265,242 unique molecules in Release 4 of the Open NCI Database [11]. When these compounds were then ranked based on their Tanimoto score, we found only two molecules whose scores indicated structural similarity: NSC 77032 (Figure 1a) and NSC 170229 (Supplementary Figure S2a) had scores of 0.65 and 0.64, respectively. All other compounds in the database search scored < 0.6. Since we had difficulty in preparing concentrated stocks of NSC 170229 in different solvents and preliminary activity assays showed that this compound was a weak inhibitor, we focused our investigation on NSC 77032 or 2-deoxy-6-phosphogluconate (2-deoxy-6-P-GA). [Note: With 6-P-F-Lys as the query, NSC 77032 and NSC 170229 were also identified as hits, albeit with Tanimoto scores of 0.59 and 0.57, respectively.]

We also applied the above approach to find compounds with structural similarity to the linear and cyclic versions of 6-P-F-Asp (the substrate of FraB). With the linear form, only one compound yielded a relatively high score: NSC 84629 (0.62, Supplementary Figure S2b). With the cyclic form of 6-P-F-Asp (Supplementary Figure S2c), the Tanimoto similarity search uncovered five structures with high similarity: NSC 78911 (0.67), NSC 57553 (0.63), NSC 206303 (0.63, a Ca^2+^ chelate of NSC 57553), NSC 351577 (0.61), and NSC 623122 (0.60) (Supplementary Figure S2d). None of these compounds have been tested for their inhibitory potential.

### 2.2. Evaluation of 2-deoxy-6-P-GA as an Inhibitor of FraB and FrlB

We sought to confirm the identity of the compound NSC 77032 as 2-deoxy-6-P-GA using NMR studies. The chemical shifts were calibrated relative to the residual HOD signal at 4.79 ppm. ^1^H-NMR (D_2_O, 600MHz) δ 4.28 (ddd, J = 8.6, 5.2, 1.7 Hz, ^1^H) H-3, 4.00 (ddd, J = 11.6, 6.7, 2.6 Hz, ^1^H, analyzed as A of ABMX) H-6, 3.96 (ddd, J = 11.9, 7.9, 4.9 Hz, ^1^H, analyzed as B of ABMX) H-6′, 3.80 (ddd, J = 8.6, 4.6, 2.7 Hz, ^1^H) H-5, 3.57 (dd, J = 8.7, 1.7 Hz, ^1^H) H-4, 2.53 (dd, J = 14.9, 8.8 Hz, ^1^H, analyzed as A of ABX) H-2, 2.47 (dd, J = 14.9, 5.1 Hz, ^1^H, analyzed as B of ABX) H-2′. The NMR spectrum of 2-deoxy-D-gluconic acid has been reported before [12].

The activities of recombinant *Salmonella* FraB and FrlB were determined using a glucose-6-phosphate dehydrogenase (G6PDH)-coupled assay [2,6,10]. We sought to obtain IC_50_ values for inhibition of FraB and FrlB from concentration-response plots (i.e., fractional activity in the presence or absence of inhibitor versus inhibitor concentration) at a single concentration of substrate (here, 1 mM). There was near-complete inhibition of FraB in the presence of 300 µM of 2-deoxy-6-P-GA. From two independent trials, the IC_50_ for inhibition of FraB by 2-deoxy-6-P-GA was determined to be 67 ± 14 µM (Figure 1b; Supplementary Figure S3). In contrast to our findings with FraB, even 500 µM of the inhibitor led to only 50% inhibition of FrlB (Figure 1b). In fact, there was partial FrlB activity even at 1 mM 2-deoxy-6-P-GA. A reliable measurement of the IC_50_ value for FrlB inhibition could not be obtained. This difference between FraB and FrlB is likely an underestimate since these IC_50_ measurements were performed using1 mM substrate, which represents an 8- and 2-fold excess over the *K_m_* for FraB and FrlB, respectively [6,10].

**Figure 2:**
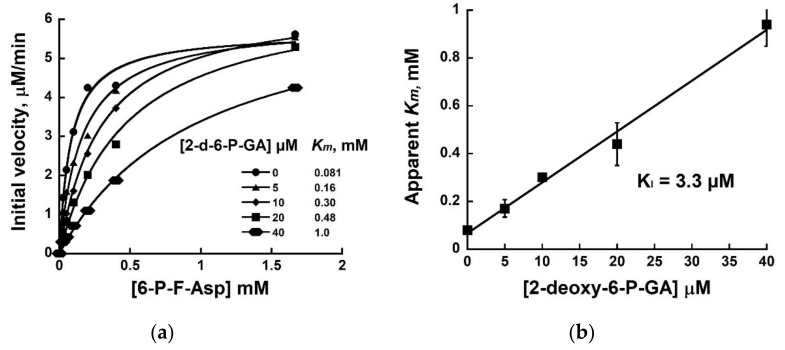
Inhibition of FraB by 2-deoxy-6-P-GA: (**a**) Representative Michaelis–Menten analyses performed in the absence or presence of 5, 10, 20, or 40 µM of 2-deoxy-6-P-GA (data from a replicate trial are provided in Supplementary Figure S4); (**b**) Plot of apparent *K_m_* values versus the inhibitor concentration to determine the inhibition constant K_I_. Error bars represent the standard error calculated from two trials.

**Figure 3:**
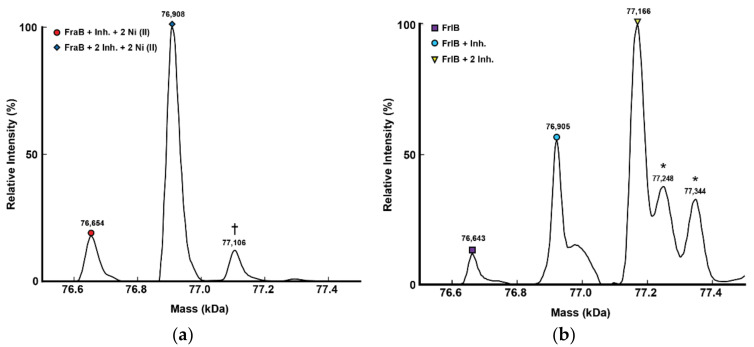
Native mass spectrometry studies to determine binding of 2-deoxy-6-P-GA to FraB (**a**) and FrlB (**b**). The protein to inhibitor ratio was 3 µM:450 µM. Each species is annotated and the observed versus expected masses are summarized in Supplementary Table S1. Extra mass (the peak indicated with † in panel (**a**)) is assumed to be due to Ni^2+^ adducts because a Ni^2+^-affinity column was used for purification of His_6_-tagged FraB. Asterisks (*) denote unannotated species. The full native (and additional) MS spectra are shown in Supplementary Figure S5. Abbreviation: Inh. refers to the inhibitor 2-deoxy-6-P-GA.

Michaelis–Menten analyses performed with FraB either in the absence or presence of four different 2-deoxy-6-P-GA concentrations showed that the *K_m_* increased with increasing inhibitor concentration, but the *V_max_* remained largely unaffected (Figure 2a). By plotting the apparent *K_m_* values versus the corresponding inhibitor concentrations used in the assay, we obtained an inhibition constant K_I_ of 3.3 µM for the inhibition of FraB by 2-deoxy-6-P-GA (Figure 3b). The K_I_ value was determined using kinetic parameters obtained from two independent trials (Figure 2a, Supplementary Figure S4).

### 2.3. Mass Spectrometry-Based Confirmation of FraB/FrlB–2-deoxy-6-P-GA Complexes

To gain direct evidence for the binding of 2-deoxy-6-P-GA to FraB and FrlB, we employed native mass spectrometry (MS). We analyzed samples generated by mixing 150 or 300 or 450 µM 2-deoxy-6-P-GA with 3 µM of FraB/FrlB (Figure 3, Supplementary Figure S5, data not shown). These titrations revealed saturable binding of the inhibitor to both FraB and FrlB, with the latter displaying weaker binding. Consistent with our previous findings that both FraB and FrlB function as homodimers [6,10], we observed predominantly dimers bound to 2-deoxy-6-P-GA. With FraB, we found a dimer that was bound to two copies of 2-deoxy-6-P-GA and two or more nickel ions adventitiously bound to the His_6_-tag that was used as an affinity purification tag (Figure 3a; 450 µM of 2-deoxy-6-P-GA + 3 µM of FraB). Some other minor species were also detected, all of which were easily accounted for (Supplementary Table S1). With FrlB(which lacks the His_6_ affinity tag), we found a dimer either free or bound to one or two copies of 2-deoxy-6-P-GA (Figure 3b; 450 µM of 2-deoxy-6-P-GA + 3 µM of FrlB). These results confirm the direct interaction of 2-deoxy-6-P-GA to FraB and FrlB. Albeit not quantitative, these MS data also suggest that the binding of 2-deoxy-6-P-GA to FraB is likely to be of higher affinity than to FrlB, an inference that is consistent with the inhibition trend and the Tanimoto similarity scores (Figure 1, Supplementary Figure S2).

### 2.4. Cell-Based Assays with Salmonella Wild-Type, ∆fra, and ∆tolC Strains

**Figure 4:**
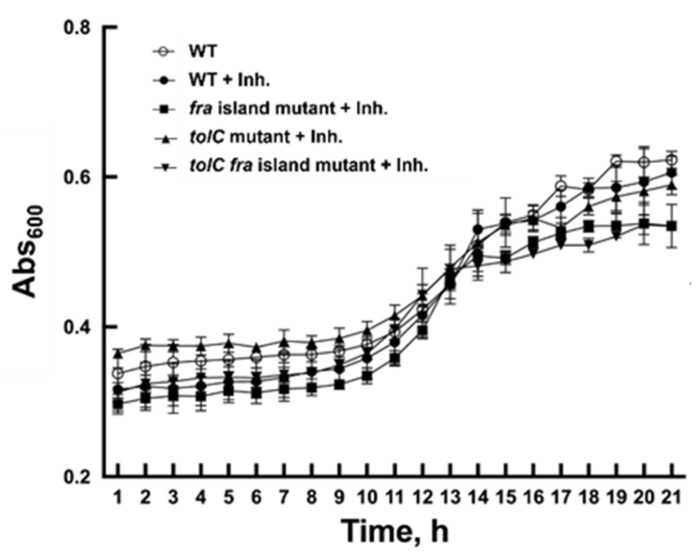
Assessing the efficacy of 2-deoxy-6-P-GA in a live-cell assay. Growth of wild-type (14028, circles), *fra* island mutant (CS1005, squares), *tolC* mutant (EFB044, triangles), and *tolC fra* island double mutant (ASD1006, inverted triangles) *Salmonella* strains in the absence or presence of 125 µM 2-deoxy-6-P-GA. Growth was measured by monitoring Abs_600_ in a Molecular Devices SpectraMax i3x. Error bars in the growth curves represent the mean and standard deviation calculated from three technical replicates associated with a single biological trial. Abbreviation: Inh. refers to the inhibitor 2-deoxy-6-P-GA.

To assess the inhibitory effect of 2-deoxy-6-P-GA in a live-cell assay, we tested in parallel a wild-type strain and a second strain (the *fra* island mutant) that lacks the entire *fra* locus. This mutant strain is unable to utilize F-Asn and form 6-P-F-Asp. Any inhibitor specific for FraB is expected to inhibit the wild-type but not the *fra* island mutant, as the latter cannot be intoxicated by 6-P-F-Asp accumulation. Both *Salmonella* strains were grown in M9 minimal medium containing 5 mM glucose and 1 mM F-Asn, and their growth was monitored hourly over a 20-h period. Neither strain showed any growth defect in the presence of 125 µM 2-deoxy-6-P-GA (Figure 4). There was no difference in this outcome when we used glycerol or fructose as the carbon source instead of glucose (Supplementary Figure S6). To test the idea that 2-deoxy-6-P-GA might be subject to efflux, we tested the inhibitor’s potency using a *tolC* mutant (wild-type or *fra* island mutant background), since *tolC* encodes a key component of most efflux systems. However, we observed no inhibition even when *tolC* is absent (Figure 4 and Supplementary Figure S6).

### 2.5. Dephosphorylation of 2-deoxy-6-P-GA by YigL

**Figure 5:**
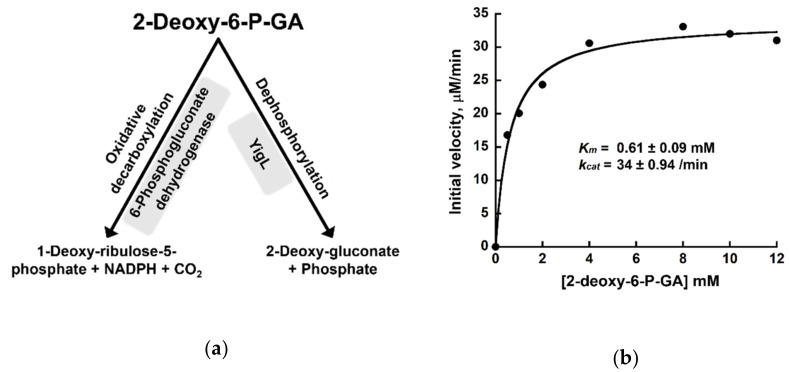
(**a**) Two different metabolic transformations of 2-deoxy-6-P-GA; (**b**) Representative Michaelis–Menten analysis for YigL-catalyzed dephosphorylation of 2-deoxy-6-P-GA. The calculated kinetic parameters and the associated curve-fit errors are shown. An additional replicate is shown in Supplementary Figure S7.

The lack of inhibitory activity of 2-deoxy-6-P-GA in the live-cell assay may be due to its polarity that limits cellular uptake or its intracellular metabolic transformation(s). We consider two possibilities for the latter postulate. First, 6-phosphogluconate dehydrogenases from sheep liver, human erythrocytes, and *Trypanosoma brucei* catalyze oxidative-decarboxylation of 2-deoxy-6-P-GA, albeit with low efficiency [13,14] (Figure 5a). Second, 2-deoxy-6-P-GA may be dephosphorylated in vivo (Figure 5a). Bacteria have numerous phosphatases belonging to the haloacid dehalogenase (HAD)-like hydrolase family [15,16]. These phosphatases play key roles including maintenance of a steady-state metabolite pool. Because the physiological substrates for these promiscuous phosphatases are small sugar-phosphate metabolites, we sought to test the idea that a phosphatase in this family may act on 2-deoxy-6-P-GA. We focused our attention on YigL [16,17], which preferentially dephosphorylates phosphorylated six-carbon sugars and was shown to attenuate the stress associated with phosphosugars [18]. The availability in our laboratory of recombinant YigL (prepared for a different research objective) was an additional practical consideration. We tested the dephosphorylation of 2-deoxy-6-P-GA by YigL using the malachite green reagent, a highly sensitive method for measuring inorganic orthophosphate. Our results demonstrate that YigL indeed dephosphorylates 2-deoxy-6-P-GA with a *K_m_* of 0.58 ± 0.03 mM and a *k_cat_* of 34 ± 0 min^−1^ (these values reflect the mean ± standard error from two replicates: Figure 5b and Supplementary Figure S7).

## 3. Discussion

In this study, we present the serendipitous discovery of a potent competitive inhibitor for *Salmonella* FraB, a deglycase and drug target. This inhibitor (2-deoxy-6-P-GA), which was identified initially based on its structural similarity to 6-P-F-Lys, was expected to dampen the activity of FrlB that acts on 6-P-F-Lys. Although the basis for the preferential inhibition of FraB over FrlB will need to await high-resolution structures of these enzymes ± their substrates (or 2-deoxy-6-P-GA), our finding attests to the value of integrating in silico and conventional HTS-based drug discovery efforts.

Contrary to our expectations, 2-deoxy-6-P-GA did not impair growth of *Salmonella* (Figure 4). Although the transport of 2-deoxy-6-P-GA into the cell may be undermined by its high polarity, its inhibitory potential (post-uptake, however weak) may also be crippled by intracellular biochemical transformations. We hypothesized that one of the factors leading to the inability of 2-deoxy-6-P-GA to inhibit cell growth may be sugar-phosphate phosphatases. Our data show that indeed YigL dephosphorylates 2-deoxy-6-P-GA, a finding that expands its substrate suite (Figure 5b) [16]. We predict that other phosphatases (e.g., YniC and Cof), whose substrate-recognition determinants mirror YigL [16], are also likely to catalyze this reaction.

Although 2-deoxy-6-P-GA was ineffective in inhibiting the growth of *Salmonella*, we envision that non-hydrolyzable isosteres may offer better prospects. We are considering chemical modifications to this lead to improve uptake and to decrease the likelihood of cellular transformations. These ideas warrant consideration given the precedence for successful use of non-hydrolysable phosphonates (e.g., fosfomycin) as antimicrobials [19].

## 4. Materials and Methods

### 4.1. Tanimoto Search for Structurally Similar Compounds

We utilized a computational method to identify compounds that are structurally similar 6-P-F-Lys (Figure 1a and Supplementary Figure S2a). For each of the 265,242 compounds available in Release 4 of the Open NCI Database [11], the structural similarity to 6-P-F-Lys was quantitated using the Tanimoto Index available in RDKit [20]. This cheminformatics tool uses the structure-data file (SDF) structure of a molecule to create 2-D circular fingerprints that contain data about the environment of each atom in the target molecule [21]. To assign a similarity score between two molecules, their fingerprints are then compared using the Tanimoto index, a reliable metric to compare molecular structures [22]. The scores range between 0 and 1, with 0 indicating no similarities between the two molecules and 1 indicating that the two molecules have the exact same structure. The 265,242 molecules were then ranked based on their similarity score to 6-P-F-Lys. The top two-scoring compounds, NSC 77032 and NSC 170229, were both available from the NCI and were subsequently ordered for experimental assays.

We also performed the same Tanimoto index search using both the linear and cyclic versions of 6-P-F-Asp (Supplementary Figure S2b,d). However, these leads were not pursued.

### 4.2. NMR Studies

2-Deoxy-6-P-GA was dried at 2 Mbar over P_2_O_5_ overnight before approximately 1 mg of the product was dissolved in 0.6 mL of D_2_O (99.8%) and transferred to a 5 mm NMR tube. The proton NMR data were collected at 298K on a Bruker Avance III-600 spectrometer equipped with a 5-mm triple-resonance inverse cryoprobe with Z-gradients. A simple zg30 pulse sequence was executed with 64 scans, 2.0 s recycle delay, 1.7 s acquisition time, 9615 Hz spectral width, and carrier frequency at water resonance. The chemical shift was referenced to the residual H_2_O signal.

We obtained 2-deoxy-6-P-GA from NCI, which does not have any information on synthesis of this compound. However, syntheses reported thus far [23,24,25] start from chiral precursors. Therefore, it is likely that our sample has the chirality that we have given, although further tests are necessary to unambiguously establish the stereochemistry. 

### 4.3. Protein Overexpression and Purification

For all the recombinant proteins used in this study, protein overexpression in *E. coli* and subsequent affinity purification was carried out as described previously [6] with some modifications. FraB was essentially purified as described before, except that we used the 325-aa construct (see ref. [6] for details). A synthetic codon-optimized construct was purchased from GeneUniversal for FrlB (cloned into pET-28b) and YigL (cloned into pET-24a). Each ORF was designed to ensure that proteins were expressed with an N-terminal His_6_-tag. For overexpression of FrlB and YigL, we used *E. coli* BL21(DE3) cells and induced protein synthesis with 1 mM IPTG for 3 h (compared to 2 h for FraB). We added 1X ProteaseArrestTM protease inhibitor cocktail (G-BioSciences, St. Louis, MO, USA) to the crude lysate to prevent proteolysis. Following purification of the recombinant FraB and YigL, the His_6_-tag was not removed (Supplementary Figure S8), largely due to the failure of TEV to cleave in between the tag and the desired proteins. However, we verified that the presence of the tag was not detrimental to YigL’s function.

### 4.4. Inhibition Assays

The activity of recombinant FrlB and FraB was evaluated in the presence or absence of 2-deoxy-6-P-GA by employing a coupled assay system that affords continuous measurement of NADH produced by glucose-6-phosphate dehydrogenase (G6PDH; Worthington Biochemical Corporation; LS003997) [6]. Glucose-6-phosphate, which is formed as a result of deglycation by either FraB or FrlB, is oxidized by G6PDH to 6-phosphoglucono-δ-lactone with concomitant reduction of NAD(P)^+^ to NAD(P)H, which can be detected by its fluorescence emission at 450 nm. For the IC_50_ measurements, the assays contained 0.2 µM enzyme, 1 mM 6-P-F-Asp/1 mM 6-P-F-Lys, 50 mM HEPES (pH 8 at 22 °C), 5 mM MgCl_2_, 0.1 mM EGTA, 1 mM NAD^+^, 10 mU G6PDH. Twenty-eight µL of the assay mix was pre-incubated with 1 µL of the compound for 25 min at 22 °C. This enzyme-inhibitor mixture was then transferred to a 384-well microplate and the deglycase reaction was initiated by adding 1 mM substrate (a substrate concentration that is nearly an order of magnitude higher than the reported *K_m_* of FraB [6] and two-fold higher in the case of FrlB [6,10]). Initial velocities were determined by monitoring the increase in the fluorescence at 450 nm. Fluorescence was measured every 3 s for a total of 2 min using a BioTek Synergy plate reader set at 37 °C (excitation at 350 nm, emission at 450 nm, and gain 100%). The rate was determined using an NADH standard curve, which was generated by plotting fluorescence versus known concentrations of NADH. For each assay, the control reaction included all components of the assay mixture except the enzyme, which was substituted with 1 µL buffer.

For determining the IC_50_ values, we used a concentration-response plot. The relative activities of FrlB and FraB were plotted versus different concentrations of the 2-deoxy-6-P-GA. At least two independent trials were conducted with FrlB and FraB (Figure 1b and Supplementary Figure S3), and the curve-fit errors did not exceed 24% in either trial. The IC_50_ values were determined using:

(1) $$y=1-\left[\frac{lx}{IC_{50}+x}\right]$$

where *y* is the relative activity (%), *x* is the inhibitor concentration (µM), and *l* is the lowest relative activity (%).

To determine the mode of inhibition and the inhibition constant (K_I_), we performed Michaelis–Menten analyses with FraB and six different concentrations of 6-P-F-Asp in the absence or in the presence of four different concentrations 2-deoxy-6-P-GA. The initial velocities were calculated as described above and the kinetic parameters (*K_m_*, *k_cat_*) were obtained by fitting the data using Kaleidagraph, version 4.5.3 for Windows, (Synergy Software, Reading, PA, USA). The curve-fit errors for *K_m_* and *k_cat_* did not exceed 24 and 13%, respectively, in any trial. The goodness of fit was determined to be ≥ 0.99 in all cases.

### 4.5. Mass Spectrometry (MS) Analyses

FraB and FrlB samples for MS analyses were prepared by buffer exchanging into 200 mM ammonium acetate (Sigma-Aldrich, St. Louis, MO, USA) using 6-kDa cutoff Micro Bio-Spin 6 Columns (Bio-Rad, Hercules, CA, USA). For FraB, the pH was adjusted to 7.4 by adding ammonium hydroxide solution (Sigma-Aldrich, St. Louis, MO, USA). A NanoDrop spectrophotometer (Thermo Scientific, Waltham, MA, USA) was used to measure the protein concentration using Abs_280_ values. The protein solutions were then diluted to the desired concentrations using 200 mM ammonium acetate. For characterization of protein-ligand complexes, 2-deoxy-6-P-GA stock solution (in water) was diluted using 200 mM ammonium acetate to the desired concentration and then mixed with protein at the indicated ratios at 25 °C. After adding the compound to the protein, samples were incubated for 30 min at room temperature before filling the capillary tip and spraying.

Native MS experiments were conducted using an in-house modified Thermo Q Exactive Ultra High Mass Range (UHMR) Orbitrap MS (Bremen, Germany). For all the experiments, a nano-electrospray ionization (nano-ESI) source was used. Approximately 2 µL of each sample was transferred into pulled borosilicate capillaries and an electrospray voltage of 0.7–0.9kV was applied to a platinum wire in contact with the solution. The mass spectrometer was operated under positive mode with the following settings: capillary temperature, 200 °C; trap gas flow rate, 5; m/z range, 600–12000. The resolution was set at either 6000 or 12,500. Mass spectra were then extracted using Xcalibur 4.1 (Thermo Scientific, Waltham, MA, USA) and processed using Unidec software tools [26] version 5.0.3

### 4.6. Live-Cell Assays

*Salmonella* strains (Supplementary Table S2) [27] were grown overnight in LB at 37 °C. Pelleted cells were washed with sterile water and resuspended in water and each strain was inoculated at a 1:10,000 dilution in a 96-well plate with M9 minimal medium containing 5 mM glucose and 1 mM F-Asn [28,29] either in the presence or absence of 125 μM 2-deoxy-6-P-GA. The plate was incubated at 37 °C for 20 h in a Molecular Devices SpectraMax i3x, with the wells covered with Breathe-Easy film (Diversified Biotech, Dedham, MA, USA) to prevent desiccation. During this incubation, the absorbance at 600 nm was measured hourly.

### 4.7. Dephosphorylation of 2-deoxy-6-P-GA by YigL

To determine the efficiency of the YigL-catalyzed dephosphorylation of 2-deoxy-6-P-GA, we employed the malachite green reagent (Sigma Aldrich, St. Louis, MO, USA)-based discontinuous assay that measures phosphate. Malachite green is a cationic dye that forms with free phosphate and molybdate a green-colored complex (Abs_620_) and enables measurement of even nanomolar quantities of inorganic orthophosphate [30]. The YigL assay contained 1 µM of recombinant His_6_-tagged-YigL, specified concentrations of 2-deoxy-6-P-GA, 25 mM Tris-Cl (pH 7.8 at 22 °C), 4 mM MgCl_2_, 0.1 mg/mL bovine serum albumin, and 1 mM dithiothreitol. Twenty-nine µL of the assay mix (not containing the substrate) was incubated at 37 °C for 3 min and the cleavage reaction was initiated by addition of 1 µL 2-deoxy-6-P-GA (to yield the desired final concentration). Two-µL aliquots of the reaction were withdrawn at five different time points and each aliquot was terminated by addition of the malachite green reagent (discontinuous assay). The quenched reaction was then incubated at 25 °C for 30 min to allow color development before measurement of Abs_620_. A standard curve was generated using Abs_620_ versus different concentrations of standard P_i_ (0 to 40 µM) and used as a reference for calculating the inorganic orthophosphate produced as the result of YigL activity. All assays were performed in duplicates. From the calculated initial velocities, we determined the kinetic parameters (*K_m_* and *k_cat_*) using Kaleidagraph to fit the data. The curve-fit errors for *K_m_* and *k_cat_* did not exceed 29% and 5%, respectively, in either trial. The goodness of fit was determined to be ≥ 0.98 in all trials.

## Data Availability

Data from replicate trials are provided in the Supplementary Materials. Any other information pertaining to this study that is not included here will be made available from the corresponding author upon request.

## Supplementary Materials

The following supporting information can be downloaded at: https://www.mdpi.com/article/10.3390/pathogens11101102/s1, Figure S1: Schematic of the pathways for bacterial uptake and catabolism of fructose-asparagine and fructose-lysine; Figure S2: Structures of hits in the NCI compound library that yielded a Tanimoto score ≥ 0.6 when queried with either 6-P-F-Lys or 6-P-F-Asp; Figure S3: Replicate IC_50_ curves showing relative percent activity of FraB and FrlB in the presence of 2-deoxy-6-P-GA; Figure S4: Replicate Michaelis–Menten curves for inhibition of FraB by 2-deoxy-6-P-GA; Figure S5: Native mass spectrometry studies to determine binding of 2-deoxy-6-P-GA to FraB and FrlB; Figure S6: Assessing the efficacy of 2-deoxy-6-P-GA in a live-cell assay in the presence of 5 mM glucose, glycerol, or fructose; Figure S7: Michaelis–Menten analysis for cleavage of 2-deoxy-6-P-GA by YigL; Figure S8: SDS-PAGE analysis of recombinant proteins used in this study; Table S1: Expected and observed masses of the proteins and protein-inhibitor complexes characterized in this study; Table S2: *Salmonella* strains used in the cell-based assays.
