# Supplementary material for: Serendipitous Discovery of a Competitive Inhibitor of FraB, a Salmonella Deglycase and Drug Target
Source: Pathogens. 2022 Sep 26;11(10):1102. doi: 10.3390/pathogens11101102 (PMC9609667; doi:10.3390/pathogens11101102)
Supplement: Supplementary file 1 [file pathogens-11-01102-s001.zip › pathogens-1882247-supplementary.pdf]

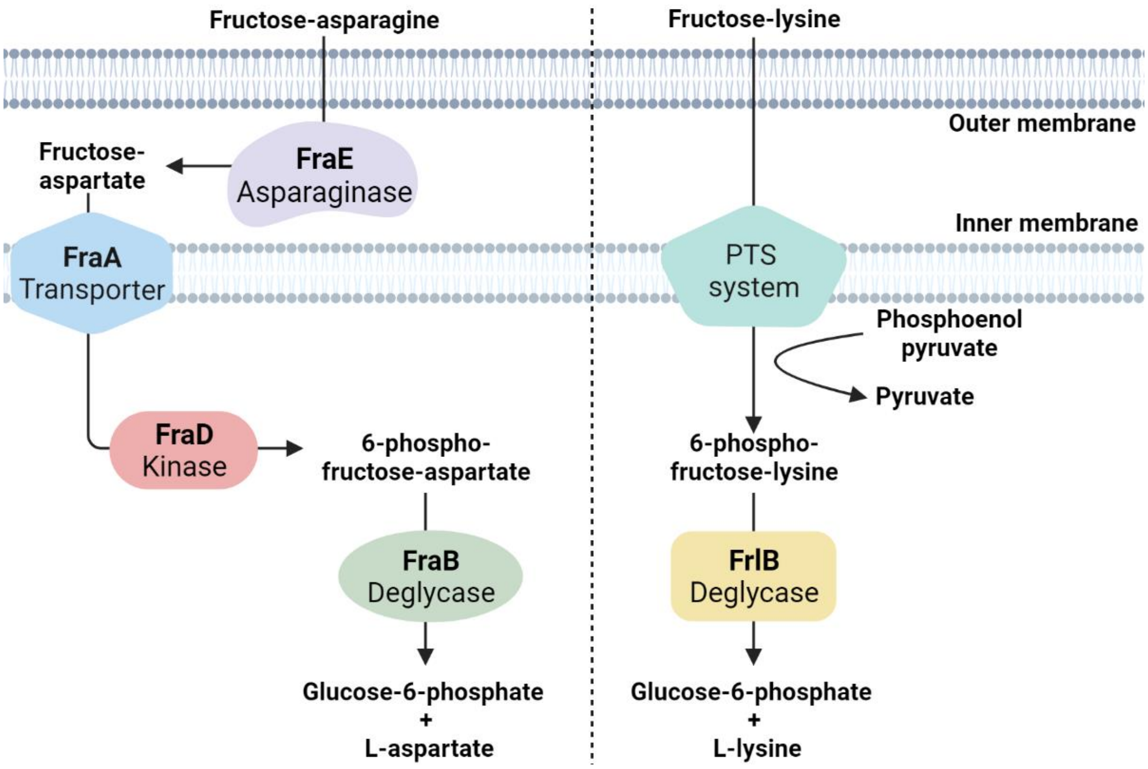

Supplementary Figure S1. Schematic of the pathways for bacterial uptake and catabolism of fructose-asparagine (left) and fructose-lysine (right) [2,5-7,9]. Some thematic variations for fructose-lysine utilization exist, but these alternatives are not depicted here.

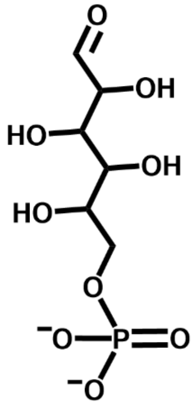

NSC 170229

(a)

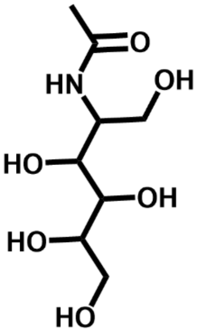

NSC 84629

(b)

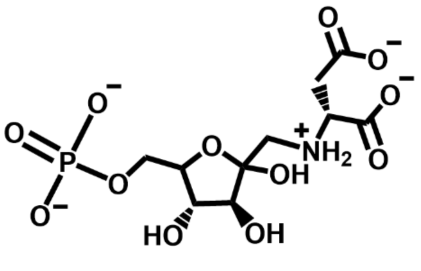

6-P-F-Asp (cyclic)

(c)

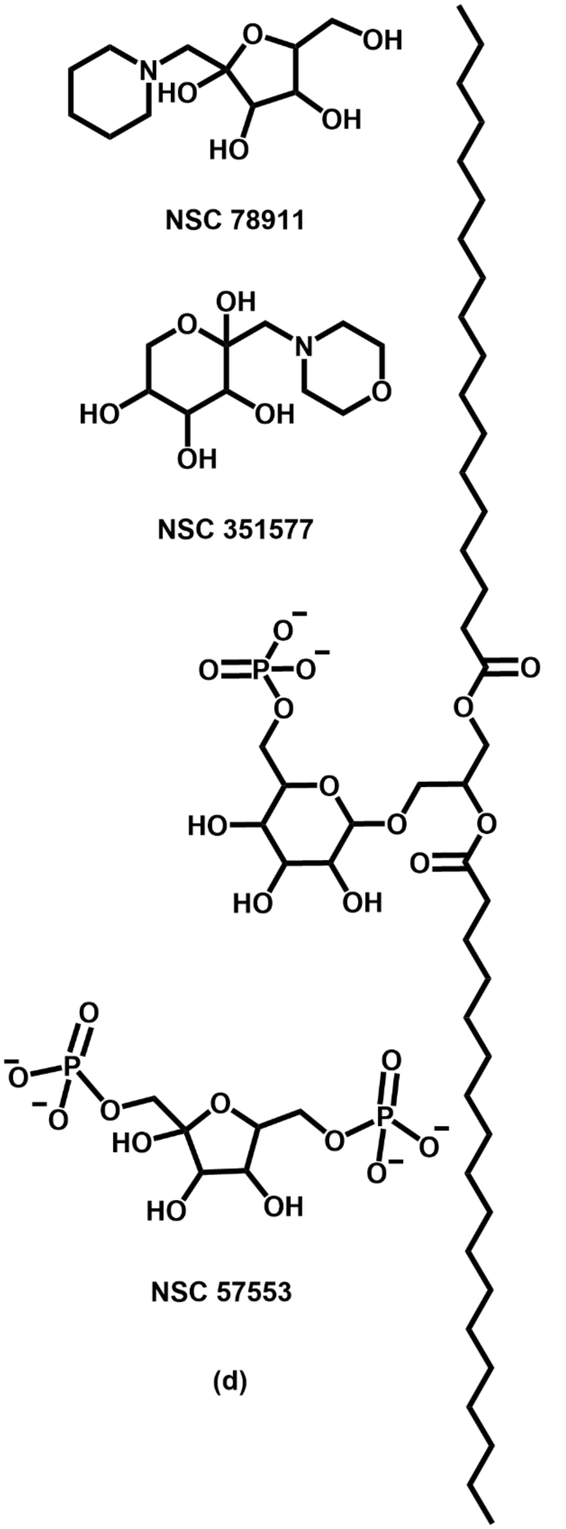

NSC 623122

(d)

Supplementary Figure S2. Structures of hits in the NCI compound library that yielded a Tanimoto score ≥ 0.6 when queried with either 6-P-F-Lys or 6-P-F-Asp. (a) When we used the Tanimoto index to assess the similarity search results

from querying 6-P-F-Lys to the NCI database, two compounds (NSC 77032 and NSC 170229, 0.65 and 0.64, respectively) had significantly higher scores than all the other molecules in the NCI database. Since the next highest score was 0.59, we chose an arbitrary cutoff score of 0.6 and used the same in future similarity searches. NSC 170229 is shown here while NSC 77032 is shown in Figure 1a. NSC 77032 and NSC 170229 were also identified when queried 6-P-F-Lys, but with scores of 0.59 and 0.57, respectively. (b) NSC 84629 was a hit against the linear version of 6-P-F-Asp with a score of 0.62; it was also identified when queried 6-P-F-Lys, albeit with a score of 0.54. This compound was not obtained and not tested in this study. (c) Chemical structure of the cyclic version of 6-P-F-Asp. (d) Compounds with a Tanimoto score > 0.6 when queried with the cyclic version of 6-P-F-Asp: NSC 78911 (0.67), NSC 351577 (0.61), NSC 57553 (0.63), and NSC 623122 (0.60). Another hit, NSC 206303, is a Ca^{2+} chelate of NSC 57553, and therefore not shown here. The stereochemistry for the NCI compounds remains to be characterized.

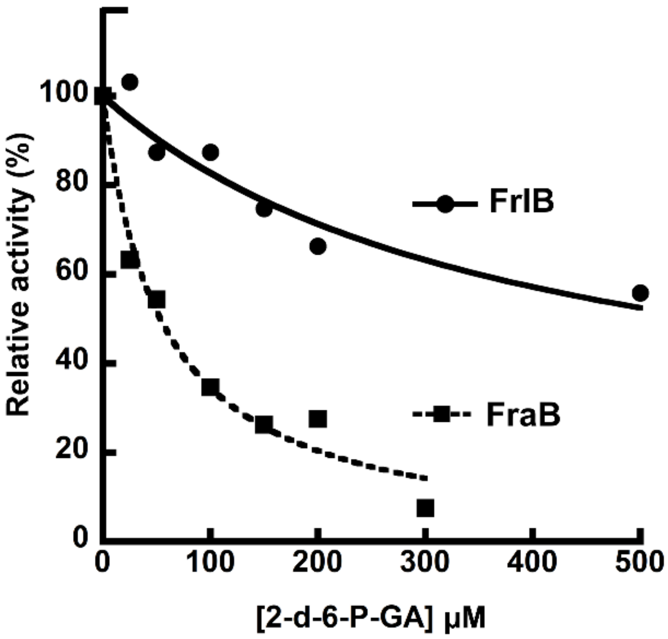

Supplementary Figure S3. Replicate IC_{50} curves showing relative activity of FraB (squares) and FrlB (circles) in the presence of 2-deoxy-6-P-GA. The goodness of fit values for the FraB and the FrlB curves are 0.99 and 0.97 respectively. These data are from trial 2 while those in Figure 1 are from trial 1.

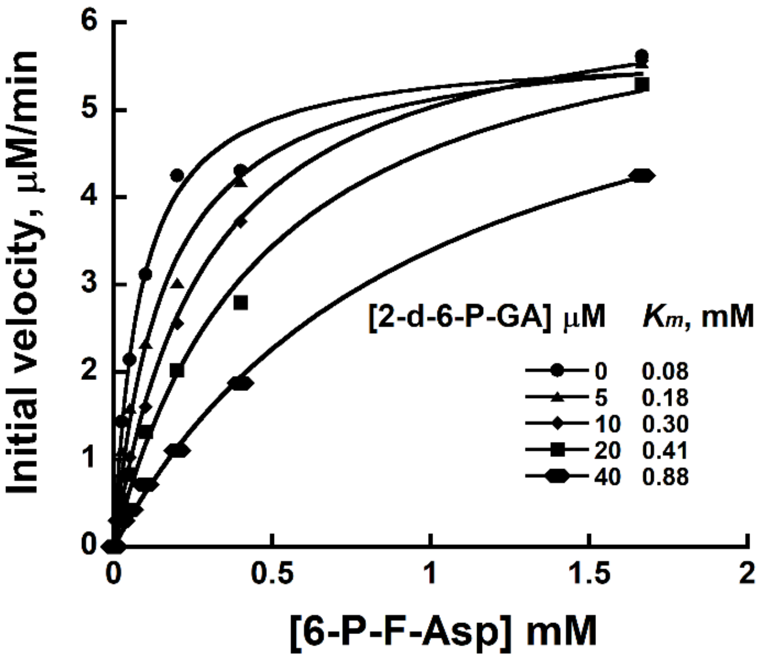

Supplementary Figure S4. Replicate Michaelis-Menten curves for inhibition of FraB by 2-deoxy-6-P-GA. These data, which are from trial 2, were used together with those from trial 1 (Figure 2a) to derive the mean ± standard error reported (Figure 2b).

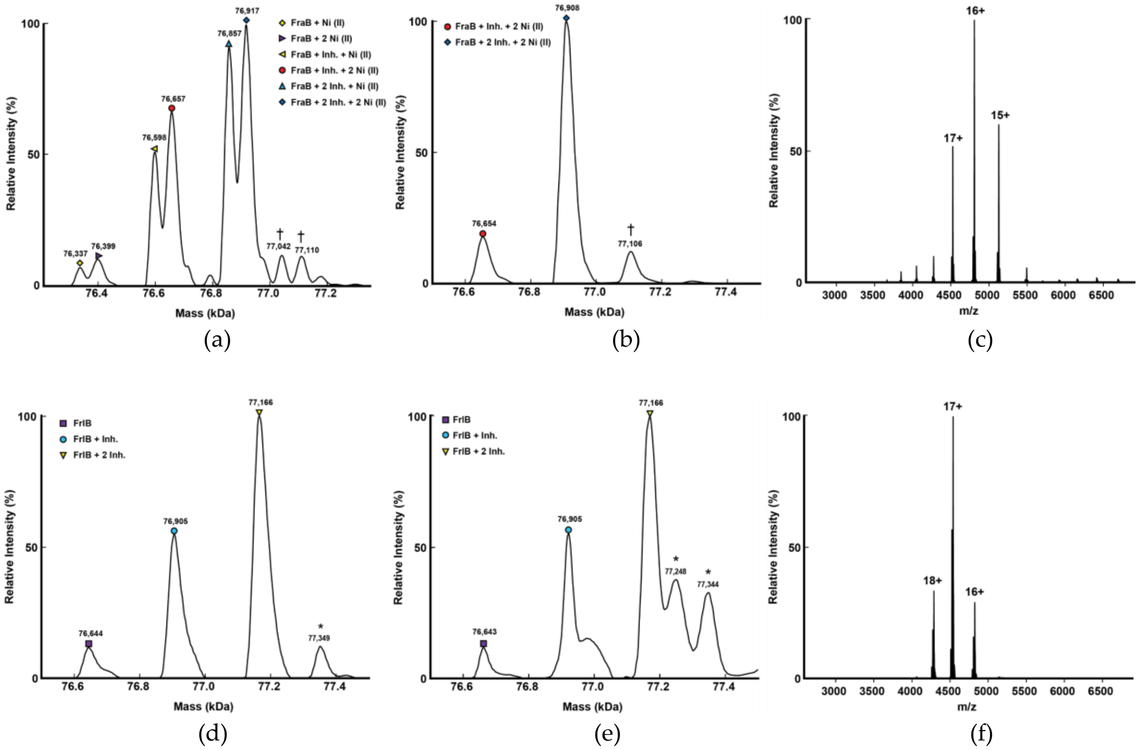

Supplementary Figure S5. Native mass spectrometry studies to determine binding of 2-deoxy-6-P-GA to FraB (a-c) and FrlB (d-f). The protein to inhibitor ratio was 3 μM:300 μM in (a) and (d), and 3 μM:450 μM in (b) and (e). The full mass spectra for the deconvolved spectra (b) and (e) are shown in (c) and (f), respectively. Abbreviation: Inh. refers to the inhibitor 2-deoxy-6-P-GA. Extra mass (the peak indicated with † in panels (a) and (b)) is assumed to be due to Ni^{2+} adducts because a Ni^{2+}-affinity column was used for purification of the His_6-tagged FraB. Asterisks (*) in panel (d) and (e) denote unannotated species.

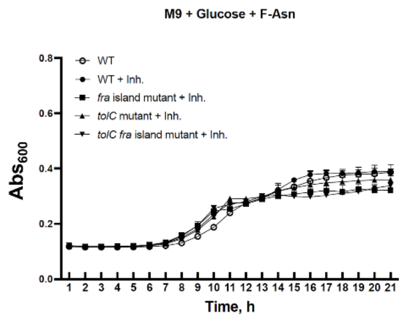

(a)

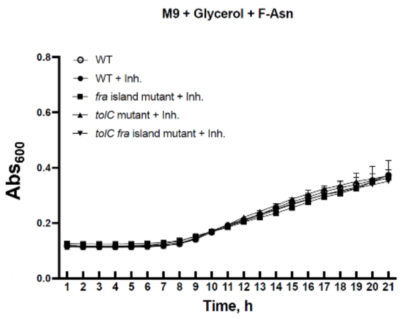

(b)

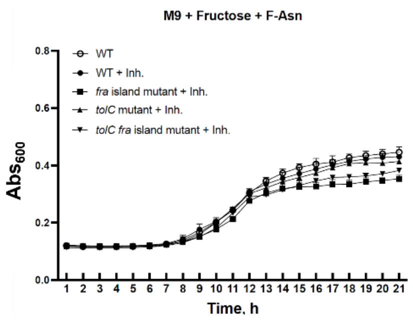

(c)

Supplementary Figure S6. Assessing the efficacy of 2-deoxy-6-P-GA in a live-cell assay in the presence of 1 mM F-Asn supplemented with 5 mM glucose (a), 5 mM glycerol (b), or 5 mM fructose (c). Growth of wild-type (14028, circles), *fra* island mutant (CS1005, squares), *tolC* mutant (EFB044, triangles) and *tolC fra* island double mutant (ASD1006, inverted triangles) *Salmonella* strains in the absence or presence of 125 μM 2-deoxy-6-P-GA. Growth was measured by monitoring Abs_{600} in a Molecular Devices SpectraMax i3x. Error bars in the growth curves represent the mean and standard deviation calculated from three technical replicates associated with a single biological trial. Abbreviation: Inh. refers to the inhibitor 2-deoxy-6-P-GA .

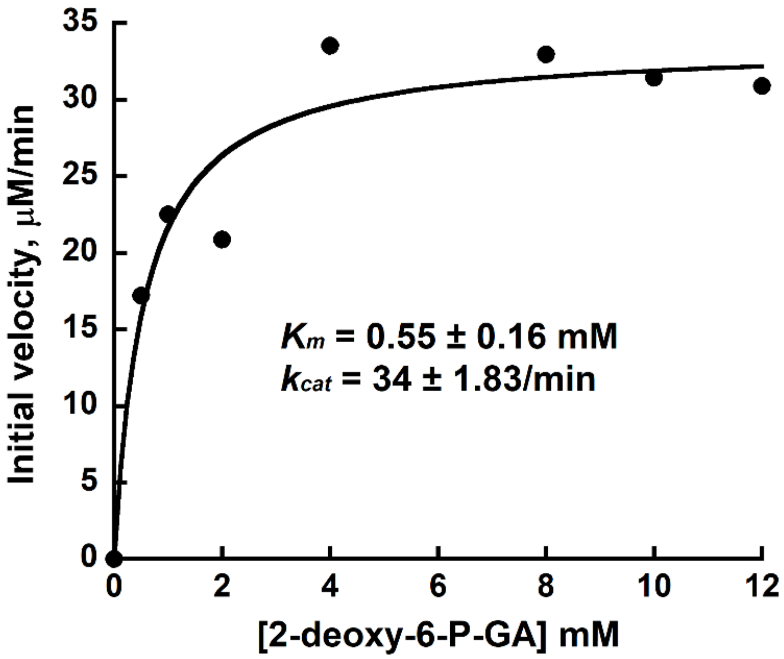

Supplementary Figure S7. Michaelis-Menten analysis for cleavage of 2-deoxy-6-P-GA by YigL. These data, which are from trial 2, were used together with those from trial 1 (Figure 5) to derive the mean ± standard error listed in the text. The calculated kinetic parameters and the associated curve-fit errors are shown.

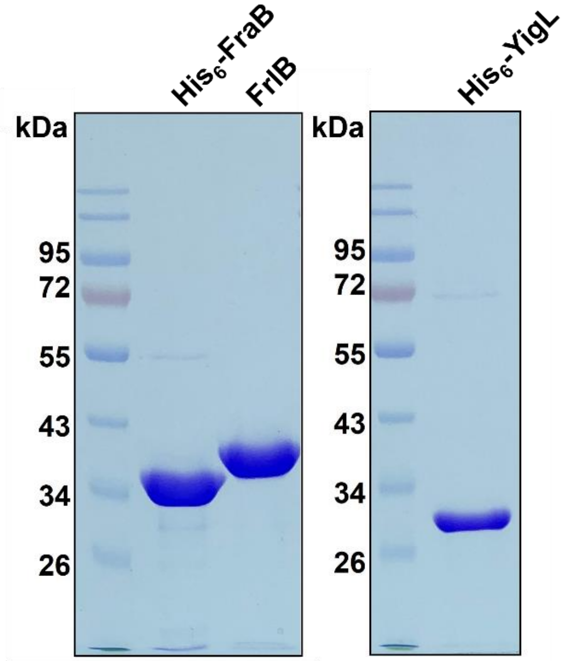

Supplementary Figure S8. SDS-PAGE [10% (w/v) polyacrylamide] analysis of recombinant proteins used in this study. The proteins loaded in each lane are indicated at the top of the gel. The PageRuler (Thermo Scientific) pre-stained protein ladder was used as reference molecular weight markers.

Supplementary Table S1

Expected and observed masses of the proteins and protein-inhibitor complexes characterized in this study

Sample	Expected mass, Da	Observed mass, Da
<u>*FraB*</u>		
His_6-FraB monomer	38,139	-
His_6-FraB monomer + Ni(II)	38,198	-
His_6-FraB dimer	76,278	-
His_6-FraB dimer + Ni(II)	76,337	76,337
His_6-FraB dimer + 2 Ni(II)	76,396	76,399
His_6-FraB dimer + 2-deoxy-6-P-GA	76,538	-
His_6-FraB dimer + 2 [2-deoxy-6-P-GA]	76,798	-
His_6-FraB dimer + 2-deoxy-6-P-GA + Ni(II)	76,597	76,598
His_6-FraB dimer + 2-deoxy-6-P-GA + 2 Ni(II)	76,656	76,654; 76,657
His_6-FraB dimer + 2 [2-deoxy-6-P-GA] + Ni(II)	76,857	76,857
His_6-FraB dimer + 2 [2-deoxy-6-P-GA] + 2 Ni(II)	76,916	76,908; 76,917
<u>*FrlB*</u>		
FrlB monomer	38,316	-
FrlB dimer	76,633	76,643; 76,644
FrlB dimer + 2-deoxy-6-P-GA	76,893	76,905
FrlB dimer + 2 [2-deoxy-6-P-GA]	77,153	77,166

Supplementary Table S2

Salmonella strains used in the cell-based assays

Strain name	Genotype	References
ATCC 14028	Wild-type *Salmonella enterica* serovar Typhimurium	American Type Culture Collection
ASD1006	14028 D*tolC::kan Δ(fraR-fraBDAE)4::Kan*	P22-mediated transduction of EFB044 into CS1005
EFB044	14028 D*tolC::kan*	P22-mediated transduction of BEI resources STM14_3859 [27] into 14028
CS1005	14028 *Δ(fraR-fraBDAE)4::Kan*	[29]
